# Editorial: Effects of autism spectrum disorder (ASD) risk genes on phenotypes of each hierarchy

**DOI:** 10.3389/fneur.2024.1508494

**Published:** 2024-10-22

**Authors:** Jun Egawa, Vance P. Lemmon, Toshiyuki Someya

**Affiliations:** ^1^Department of Psychiatry, School of Medicine, Niigata University, Niigata, Japan; ^2^Graduate School of Medical and Dental Sciences, Niigata University, Niigata, Japan; ^3^Miami Project to Cure Paralysis, The University of Miami Miller School of Medicine, Miami, FL, United States

**Keywords:** autism spectrum disorder, *Trio* gene, *PANK2* gene, sensory processing, social isolation, phenotypic hierarchies

**Figure 1:**
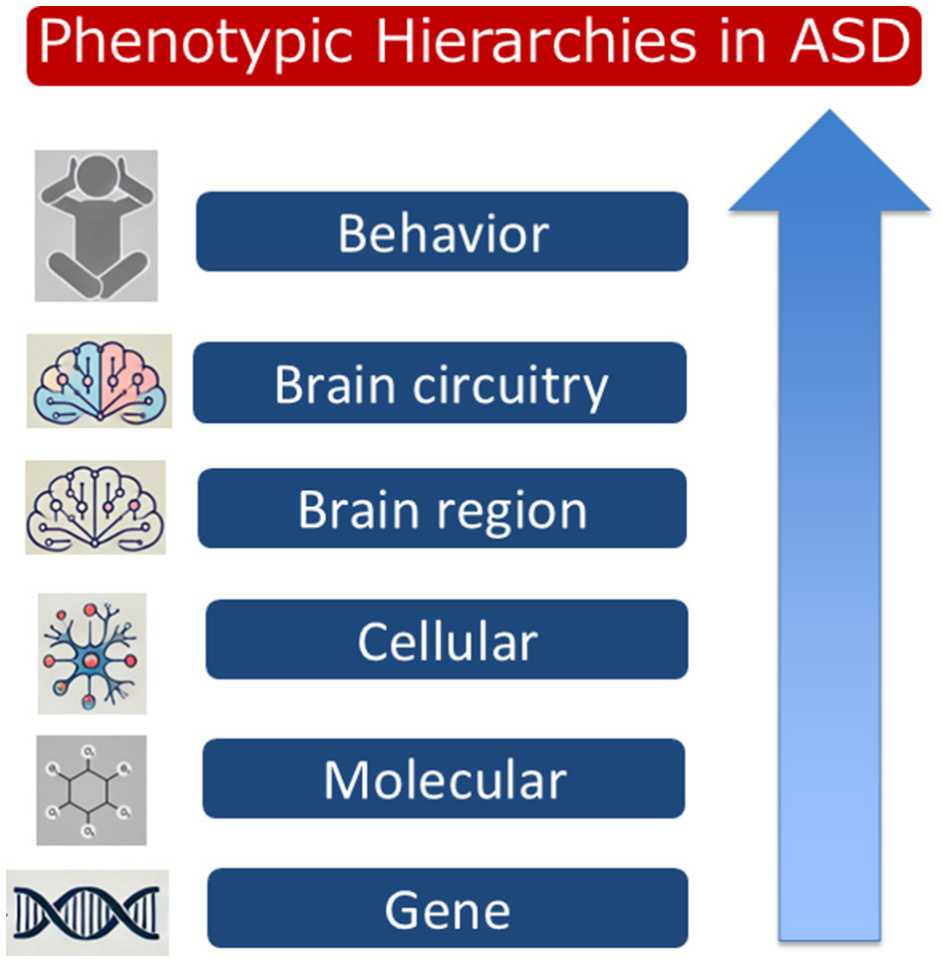
A hierarchical representation of phenotypic impacts in Autism Spectrum Disorder (ASD). The diagram illustrates the relationship between different levels, from molecular and cellular alterations to gene expressions, brain regions, brain circuitry, and resulting behavioral phenotypes. This hierarchy emphasizes the multi-level approach needed to understand the diverse manifestations of ASD.

Autism spectrum disorder (ASD) is a highly complex neurodevelopmental disorder characterized by a broad range of behavioral and cognitive manifestations. Genetic research has identified ~1,000 risk genes linked to ASD. Many of these genes are involved in critical processes of neuronal development, such as synapse formation, axonal growth, and synaptic pruning during the prenatal and early postnatal stages. However, the exact contributions of these risk genes to the clinical heterogeneity observed in ASD remain poorly understood. This problem is further compounded by risk genes altering molecular functions and cellular processes that change brain circuitry and, ultimately, behavior (Figure 1). Given this complexity, understanding how individual risk genes influence phenotypes at successive hierarchical levels is essential for advancing ASD research and therapeutic development.

The Research Topic *Effects of autism spectrum disorder (ASD) risk genes on phenotypes of each hierarchy* was conceived to address these gaps. This Research Topic of studies focuses on elucidating the hierarchical impact of ASD risk genes by examining their effects at various levels, including molecular, cellular, circuit, and behavioral phenotypes. By investigating how changes at each level contribute to the broader ASD phenotype, these studies aim to identify convergent pathways that may underlie the disorder's diverse presentations. This editorial highlights four studies that exemplify this approach, each shedding light on different hierarchical levels and offering insights into potential therapeutic targets.

The study by Wang et al. investigates the role of the *Trio* gene in motor impairments associated with ASD. Using a mouse model with conditional *Trio* deletion in cerebellar Purkinje cells (PCs), the authors found that *Trio* deficiency resulted in delayed-onset motor dysfunctions, accompanied by significant changes in the expression of essential proteins, such as Calbindin, and abnormal MRI findings. Their work reveals that motor dysfunctions in ASD can have a delayed onset depending on the genetic context, suggesting that therapeutic windows for intervention may be longer than previously thought. Moreover, this study highlights the cerebellum's role in ASD-related motor dysfunctions and suggests that targeted modulation of *Trio*-related pathways could be a viable strategy for alleviating motor symptoms (Wang et al.).

In a clinical context, Dong et al. provide a detailed case report of a young Chinese patient with compound heterozygous mutations in the *PANK2*, which is typically associated with pantothenate kinase-associated neurodegeneration (PKAN). Interestingly, this patient presented with ASD and ADHD-like symptoms, including speech difficulties, psychiatric symptoms, and mild developmental delays, which complicated the diagnostic process. Their findings emphasize the phenotypic heterogeneity of *PANK2* mutations and the importance of comprehensive genetic testing in ASD patients with atypical presentations. By revealing how disruptions in mitochondrial function can manifest as ASD-like symptoms, this study adds to the growing evidence that metabolic pathways may play a role in the pathogenesis of ASD (Dong et al.).

Monday et al. contribute a comprehensive review focusing on sensory dysfunction in ASD. They analyze multiple ASD mouse models and propose that while distinct genetic mutations can produce diverse circuit-level abnormalities, they often converge at the level of sensory processing, leading to either heightened sensory detection or impaired sensory discrimination. This dichotomy suggests that ASD may consist of two broad sensory phenotypes, which could serve as a basis for categorizing ASD subtypes. For example, many models identified increased excitation-inhibition (E-I) ratios and parvalbumin (PV) interneuron hypofunction as common circuit-level disruptions. These findings indicate that targeting sensory processing mechanisms, particularly in the auditory and visual cortices, could help alleviate core sensory and behavioral deficits in ASD (Monday et al.).

Finally, Yamaguchi et al. investigate the effects of social isolation during critical developmental windows on the prefrontal cortex (PFC) and its implications for ASD. Using a mouse model, they show that juvenile social isolation results in lasting alterations in the excitatory-inhibitory balance within the PFC, explicitly affecting the expression of neuronal activity-regulated pentraxin (NARP) and PV. These changes were associated with impaired social memory. They heightened anxiety-like behaviors in adulthood, providing a model for understanding how early environmental factors interact with genetic vulnerabilities to shape ASD phenotypes. Notably, the authors found that human lymphoblastoid cell lines derived from ASD patients also exhibited reduced NARP expression, suggesting that NARP could serve as a biomarker for specific ASD subtypes. Their findings underscore the critical role of the PFC in social and cognitive dysfunctions observed in ASD and suggest that interventions targeting early-life social environments could modulate long-term outcomes (Yamaguchi et al.).

Together, these four studies highlight the value of a hierarchical approach to studying ASD risk genes. By examining how risk genes impact phenotypes at multiple levels—from proteins and cells to circuits and behaviors—these studies contribute to a more nuanced understanding of the biological underpinnings of ASD. Such research is crucial for the development of precision therapies that can address the specific needs of ASD subgroups. For instance, while targeting molecular pathways like *Trio* or *PANK2* may benefit some individuals, others may require interventions that normalize circuit-level dysfunctions or modulate early-life environmental factors.

The implications of these findings extend beyond ASD. Many neurodevelopmental disorders, such as schizophrenia and ADHD, share overlapping genetic and phenotypic features with ASD, suggesting that insights gained from ASD research could inform the broader field of neurodevelopmental disorders. Future studies should focus on integrating data across hierarchical levels to create a comprehensive framework for understanding the complex relationships between genetic risk, neurodevelopmental processes, and behavior. Additionally, cross-species research using animal models and human subjects will be essential for validating and translating these findings into clinical practice.

In conclusion, this Research Topic has highlighted the diverse effects of ASD risk genes across hierarchical phenotypes. By taking a multidisciplinary approach, the studies in this Research Topic provide new perspectives on how genetic and environmental factors interact to shape the ASD phenotype. We hope that this Research Topic will stimulate further research into the hierarchical impact of ASD risk genes, ultimately paving the way for the development of more effective and targeted therapeutic strategies.

